# The influence of maternal body mass index and physical activity on select cardiovascular risk factors of preadolescent Hispanic children

**DOI:** 10.7717/peerj.6100

**Published:** 2018-12-13

**Authors:** Basil A. Alhassan, Ying Liu, Deborah Slawson, Jonathan M. Peterson, Jo-Ann Marrs, William A. Clark, Arsham Alamian

**Affiliations:** 1 Department of Biostatistics and Epidemiology, College of Public Health, East Tennessee State University, Johnson City, TN, United States of America; 2 Department of Community and Behavioral Health, College of Public Health, East Tennessee State University, Johnson City, TN, United States of America; 3 Department of Health Sciences, College of Public Health, East Tennessee State University, Johnson City, TN, United States of America; 4 College of Nursing, East Tennessee State University, Johnson City, TN, United States of America; 5 College of Clinical and Rehabilitative Health Sciences, East Tennessee State University, Johnson City, TN, United States of America

**Keywords:** Obesity, Elevated blood pressure, Overweight, TV screen time, Hispanic children, Physical activity

## Abstract

**Background:**

Maternal obesity and physical inactivity have been identified as correlates of overweight and obesity and physical inactivity in older preadolescents; however, no study has explored this relationship in Hispanic preadolescents. Furthermore, the relation between maternal physical activity (PA) and blood pressure (BP) in Hispanic preadolescents has not been examined.

**Purpose:**

This study aimed to assess the associations between Hispanic mothers’ PA and body mass index (BMI) and their preadolescents’ PA, screen time, BP, and BMI.

**Methods:**

Data of 118 mother-child (aged 2–10 years) dyads enrolled in a cross-sectional study of metabolic syndrome in Hispanic preadolescents at a community health center in Johnson City, TN were used. Parent and child questionnaires were used to ascertain mothers’ BMI and PA and preadolescents’ PA and screen time. Preadolescents’ height, weight, and BP were measured. Multiple logistic regression was used to examine the association between child and maternal variables, adjusting for mother’s education and the child’s sex and age.

**Results:**

Pradolescents of obese mothers were more likely than preadolescents of mothers with normal weight to engage in less than three days of at least 60 min of vigorous PA per week (OR: 6.47, 95% CI [1.61–26.0]). Preadolescents whose mothers did not engage in moderate PA were more likely to engage in less than three days of at least 60 min of vigorous PA per week (OR: 2.92, CI [1.18–7.24]); and have elevated BP (OR: 2.50, 95% CI [1.02–4.53]) than preadolescents whose mothers engaged in moderate PA.

**Discussion:**

Our results show a negative relationship between maternal obesity and preadolescent PA, and a positive relationship between lower maternal PA and elevated BP and lower PA in Hispanic preadolescents. This suggests that interventions aimed at improving Hispanic preadolescents’ PA and BP may use maternal PA and maternal BMI (for preadolescent PA) as a modification strategy to improve health in Hispanic preadolescents.

## Introduction

Physical inactivity, elevated blood pressure (BP), and overweight and obesity track from childhood to adulthood (McGill, McMahan & Gidding, 2008; Napoli et al., 2006), and increase the risk of cardiovascular diseases such as heart disease and stroke (Mozaffarian et al., 2016). According to the 2008 Physical Activity Guidelines for Americans (U.S. Department of Health and Human Services, 2008), children should engage in at least one hour of physical activity (PA) per day, including at least three days of moderate-to-vigorous intensity aerobic activity. Adequate PA in childhood can prevent obesity and elevated blood pressure (Expert Panel on Integrated Guidelines for Cardiovascular Health and Risk Reduction in Children and Adolescents, 2011). Despite evidence of the cardiovascular benefits of adequate PA and the detrimental effects of a sedentary lifestyle, a significant number of U.S children and adolescents do not meet national recommendations. Per the 2013 Youth Risk Behavior Survey (Kann et al., 2014), 15.2% of high school students nationwide did not meet the recommendation of at least 60 min of PA in the past week; 41.3% spent three or more hours playing computer or video games un-related to school work; and 32.5% spent three or more hours watching television per day. Only 27.1% of high school students nationwide met the PA guidelines. Of students in kindergarten through the 12th grade nationwide, only 29.4% attended structured daily physical education classes in school (Kann et al., 2012).

Hypertension in children is defined as average systolic (SBP) and/or diastolic (DBP) blood pressure higher than or equal to the 95th percentile for sex, age, and height on three or more repeated measurements (National High Blood Pressure Education Program Working Group on High Blood Pressure in Children and Adolescents, 2004). Elevated BP, or prehypertension, refers to average SBP or DBP higher than or equal to the 90th percentile, but less than the 95th percentile. Although from 2003–2004 to 2011–2012, the prevalence of elevated BP among 8–17-year-old U.S children decreased from 3.1% to 1.6%, the prevalence of elevated BP was 11.5% for Hispanic children versus 9.4% for non-Hispanic White children in 2011–2012 (Kit et al., 2015). Further, about eighteen percent (17.7%) of children 12-to-19 years old have either poor or intermediate scores on the American Heart Association’s BP metric of cardiovascular health (Mozaffarian et al., 2016). Routine measurement of BP during health care visits is recommended for children three or more years old (Chobanian et al., 2003; Expert Panel on Integrated Guidelines for Cardiovascular Health and Risk Reduction in Children and Adolescents, 2011).

Overweight and obesity remain a major public health problem. Childhood overweight and obesity prevalence rates are high across all racial groups; however, Hispanic children experience higher prevalence of obesity (Falkner & Cossrow, 2014; Kit et al., 2015; Nguyen, Kit & Carroll, 2015) as compared to their non-Hispanic White peers. Hispanic children are also more likely to experience earlier onset of obesity and be severely obese (Mozaffarian et al., 2016) as compared to their non-Hispanic White peers. From 2003–2004 to 2013–2014, the prevalence of obesity among 2–19-year-old U.S children decreased slightly from 17.1% to 16.9%, among 2–19-year-old Mexican American females, it increased from 16.1% to 24.2%, and among 2–19-year-old Mexican American males, it decreased from 22.0% to 19.5% (Cheryl, Carroll & Ogden, 2016). Recommended strategies to prevent obesity include programs to increase PA in the community, increase consumption of fruits and vegetables, environmental approaches to improve transportation and use of land, as well as early care and education at schools (Centers for Disease and Prevention, 2018).

A positive energy balance resulting from a set of risk factors including poor diet, physical inactivity, genes, aging and ethnicity underlie the development of overweight and obesity (Kelly et al., 2013). A similar set of risk factors including obesity, high dietary salt intake, male gender, older age, and ethnicity, drive the development of hypertension in preadolescents (Kelly et al., 2013). In addition to these established risk factors, many studies (Fuemmeler, Anderson & Mâsse, 2011; Ruiz et al., 2011; Whitaker et al., 1997) have found an association between maternal PA and body mass index (BMI), and obesity and hypertension in older children (Durand et al., 2011; Gordon-Larsen et al., 2006; Rosendranz & Dzewaltowski, 2011; Sallis et al., 2009a; Sallis et al., 2009b; Van Den Berg et al., 2013). However, no study has examined the influence of mother’s PA or BMI on Hispanic preadolescents’ BMI, PA and BP.

Therefore, this study tests two related hypotheses: First, mothers who self-report to be less physically active are less likely to self-report that their preadolescent children are physically active, and that their preadolescent children are more likely to self-report as having more screen time. Second, an increased maternal weight is associated with increased preadolescent weight and BP in Hispanic children. It is important to understand the relation between the maternal and child health characteristics among Hispanics in order to inform the development of public health interventions for the large and growing Hispanic community in Tennessee.

## Materials & Methods

### Data source and participants

Data for this study came from a cross-sectional pilot study of metabolic syndrome in Hispanic preadolescents, ages 2–10 years, who presented for well-child care at a community health center in Johnson City, TN, from June 2015 to June 2016, accompanied by their mothers. No participant in the original study reported a pre-existing diagnosis of metabolic syndrome. The study was reviewed and approved by the Institutional Review Board of the East Tennessee State University (IRB#: 0414.16s).

Of the original 150 preadolescents, 21 of a set of 21 pairs of siblings and four of a set of two sets of three siblings were randomly eliminated. Siblings were included in the initial sample because basic science researchers on the multidisciplinary team were interested in studying siblings. Two preadolescents with BMI below the fifth percentile were also excluded because of this study’s focus on normal BMI, overweight and obesity. An additional five preadolescents who did not have BMI and/or BP data were removed. The final analytic sample consisted of 118 preadolescents.

### Data collection methods

A pediatric nurse identified potentially eligible mother-child dyads two days before the well-child visit. Inclusion criteria for preadolescents were: being 2–10 years of age; Hispanic, as defined by the U.S. Census Bureau; and not having a serious physical or mental illness. Mother-child dyads were provided written and oral information on the study protocols and purpose and thereafter were requested to provide written voluntary informed consent. Children seven years or older and mothers were required to provide written informed consent to proceed with the study.

An experienced research assistant, proficient in written and spoken English language and Spanish, was trained in using a set of child and parent questionnaires to collect child and parent sociodemographic, PA, and screen time data. Mother’s height and weight were reported in the parent questionnaire. Questionnaires were administered in English or Spanish language and the mother was the respondent. A pediatric nurse practitioner measured preadolescent’s height, weight and BP using standard protocols (Centers for Disease Control and Prevention, 2007). A standard scale which was tested and calibrated daily for accuracy was used to measure weight to the nearest 0.2 pounds; a stadiometer was used to measure height to the nearest one-eighth of an inch; and auscultation with a stethoscope and a standard clinical mercury sphygmomanometer was used to measure child BP after the child rested for at least five minutes.

### Outcome measures

Child systolic and diastolic BP percentiles were obtained from CDC blood pressure charts and categorized as: 1. normal BP (systolic or diastolic BP < 90th percentile) and 2. elevated BP (systolic or diastolic BP ≥ 90th percentile) (National High Blood Pressure Education Program Working Group on High Blood Pressure in Children and Adolescents, 2004). Child BMI percentiles were calculated using the 2000 CDC growth charts (Centers for Disease Control and Prevention, 2000). Participants were grouped as: 1. underweight (less than 5th percentile), 2. Healthy weight (5th–84th percentiles), 3. Overweight (≥85th–94th percentiles), and 4. obese (≥95th percentile) (Centers for Disease Control and Prevention, 2000). Because of the study’s small sample size, the remaining three BMI categories were collapsed into two categories: 1. normal BMI preadolescents (5th through 84th percentile) and 2. Overweight and obese preadolescents which included preadolescents with BMI from 85th percentile and above, for age and sex.

Child PA was assessed by the question: “*during the past 7 days, on how many days was your child physically active for a total of at least 60 min per day? Add up all the time he/she spent in any kind of physical activity that increased his/her heart rate and made him/her breathe hard some of the time”* (National Center for Health Statistics, 2013). The responses ranged from 0 to 7 days. We categorized the number of days of preadolescents’ PA in a week using recommended guidelines (U.S. Department of Health and Human Services, 2008); however, because of the small sample size and distribution of responses, the variable was categorized as: 1. <3 days of vigorous PA per week and 2. ≥3 days of vigorous PA per week.

Child screen time (TV or video) was assessed by the question: *“over the past 30 days, on average how many hours per day did your child sit and watch TV or videos*” (National Center for Health Statistics, 2013). The responses ranged from 0 to 8. Based on the American Academy of Pediatrics’ recommendation, the variable was categorized as: 1. ≤2 h per day and 2. >2 h per day (American Academy of Pediatrics, 2010).

### Mother’s BMI and physical activity

Mother’s BMI was calculated and categorized as: 1. healthy (18.5 kg/m^2^ ≤ BMI ≤ 24.9 kg/m^2^); 2. Overweight (25 kg/m^2^ ≤ BMI ≤ 29.9 kg/m^2^); and 3. Obese (BMI ≥ 30 kg/m^2^) (Centers for Disease Control and Prevention, 2015). Mother’s PA was assessed by the question: “*in a typical week, do you do any moderate-intensity sports, fitness, or recreational activities which cause a small increase in breathing or heart rate such as brisk walking, bicycling, swimming, or golf for at least 10 min continuously?”* (National Center for Health Statistics, 2013). The responses were: 1. Yes or 2. No.

### Sociodemographic measures

Child age was computed as completed years from reported date of birth. Child sex was reported as either male or female. Age was categorized as: 1. 2–5 years; and 2. 6–10 years to reflect developmental, biological and social differences by age. Educational attainment has been used as a proxy for socioeconomic status (Hendrie et al., 2013) because income levels increase with higher educational attainment. Mother’s education was used as a proxy for mother’s socioeconomic status and categorized as: 1. less than 9th grade; 2. 9th-11th grade; 3. high school graduate/GED or equivalent; and 4. some college, Associate Academic degree or above.

### Statistical analyses

Chi-squared and Fisher’s exact tests were used to examine differences in the prevalence of preadolescents’ elevated BP, being overweight or obese, excessive screen time, and low PA levels by sociodemographic characteristics, as well as the relationship between mother’s BMI status and PA levels, and preadolescents’ elevated BP, being overweight or obese, excessive screen time, and low PA levels. Chi-squared and Fisher’s exact tests were also used to examine bivariate associations between preadolescents’ BP, BMI status, screen time, and PA. Independent variables with a *P* value < 0.20 in univariate associations were entered in multiple logistic regression models of child’s low PA levels, being overweight or obese, and elevated BP (Mickey & Greenland, 1989; Fagerland, Hosmer & Bofin, 2008). Variables with a *P* ≥ 0.20 were also entered one by one in the multivariable model to identify confounders. Child age, child sex, as well mother’s education were included in all models as they are sociodemographic variables universally considered as potential confounders. Alpha less than 0.05 was set as the threshold for statistically significant associations. Data analysis was performed in statistical analyst system (SAS version 9.4; SAS Institute, Cary, NC, USA). Outcome and independent variables were treated as categorical variables for ease of interpreting results from logistic regression analysis. Regrouping within each variable was necessitated by the small sample size of this study.

## Results

All mothers and children in the study identified themselves as Hispanic. The mean age of the preadolescents was 6.36 years (*SD* = 2.75) and half were female (Table 1). Children subjects were identified as 76.3% Mexican-American, and 23.7% as of other Hispanic origin (Puerto Rican, Guatemalan, Ecuadorian and Columbian). About eighty nine percent (88.9%) of mothers had no education beyond high school or the equivalent of high school (Table 1).

**Table 1:** Sociodemographic characteristics of preadolescents by cardiovascular risk factors (*N* = 118).^a^

Characteristic, *n* (%)	*n* (%) Total	Elevated BP *n* (%)		Being overweight/ obese *n* (%)		>2 h Screen time^b^/ day *n* (%)		<3 Days PA/ Wk *n* (%)	
			*p*-value^c^		*p*-value^c^		*p*-value^c^		*p*-value^c^
***n* (%) Total**	118(100.0)	37(31.4)		48(40.7)		26(22.0)		31(26.3)	
**Sex, *n* (%)**			0.17		0.45		0.66		0.83
Male	59(50.0)	22(37.3)		26(44.1)		14(23.7)		16(27.1)	
Female	59(50.0)	15(25.4)		22(37.3)		12(20.3)		15(25.4)	
**Age group, *n* (%)**			0.007		0.47		0.33		0.70
2–5 years	46(39.0)	21(45.7)		18(39.1)		8(17.4)		13(28.3)	
6–10 years	72(61.0)	16(22.2)		30(41.7)		18(25.0)		18(25.0)	
**Mother’s education, *n* (%)**			0.95		0.93		0.10		0.27
<9th grade	53(44.9)	18(34.0)		23(43.4)		12(22.6)		14(26.4)	
9–11th	20(16.9)	6(30.0)		7(35.0)		4(20.0)		2(10.0)	
High school/GED	32(27.1)	9(28.1)		13(40.6)		4(12.5)		11(34.4)	
≥Some college	13(11.0)	4(30.0)		5(38.5)		6(46.2)		4(30.8)	
**Hispanic Origin, *n* (%)**			0.57		0.86		0.67		0.19
Mexican-American	90(76.3)	27(30.0)		37(41.1)		19(21.1)		21(23.3)	
Other^d^	28(23.7)	10(35.7)		11(39.3)		7(25.0)		10(35.7)	

**Notes.**

a Data from a study of metabolic syndrome in Hispanic children, at a community health center in Johnson City, TN, June 2015–June 2016.

b Screen time: includes time watching TV and videos.

c *p*-value from chi-squared or Fisher’s exact test.

d Other Hispanic origin (Puerto-Rican, Argentine, Columbian, Guatemalan, Argentine, Ecuadorian and El-Salvadorian).

Abbreviations BP Blood Pressure GED General Education Development >2 h Screen/day greater than an average of 2 h of watching TV or videos in the past 30 days <3Days PA/Wk Less than 3 days of physical activity for at least 60 min per day in the past 7 days

About a third (31.4%) of preadolescents had elevated BP, and about 4 out of 10 (40.7%) were overweight or obese. About a fifth (22.0%) of preadolescents spent over two hours per day watching TV or videos, and just over a quarter (26.3%) engaged in less than three days of sixty or more minutes of vigorous PA per week (Table 1).

Two-to-five-year-old preadolescents had a higher prevalence of elevated BP compared to 6-to-10-year-olds (45.7% vs. 22.2%, *p* = 0.007) (Table 1). Likewise, preadolescents whose mothers did not engage in moderate PA tended to have a higher prevalence of elevated BP (38.7% vs. 23.2%, *p* = 0.07) (Table 2); and higher prevalence of engaging in less than three days of at least 60 min of vigorous PA per week, (33.9% vs. 19.4%, *p* = 0.072) (Table 2), than preadolescents whose mothers engaged in moderate PA. Preadolescents whose mothers did not engage in moderate PA tended to have a higher prevalence of being overweight/obese than preadolescents whose mothers engaged in moderate PA, (46.8% vs. 33.9%, *p* = 0.08). Table 3 shows bivariate associations between preadolescent’s PA, screen time, BP and BMI. Preadolescents’ BMI and BP were significantly associated (*p* = 0.046).

**Table 2:** Maternal body mass index and physical activity and preadolescents’ cardiovascular risk factors (*N* = 118).^a^

Characteristic (*n* %)	*n* (%) Total	Elevated BP *n* (%)		Being overweight/ Obese *n* (%)		>2 h Screen^b^/ day *n* (%)		<3 Days PA/ Wk *n* (%)	
			*p*-value^c^		*p*-value^c^		*p*-value^c^		*p*-value^c^
**Moderate PA by mother, *n* (%)**			0.07		0.08		0.551		0.072
Yes	62(52.5)	13(23.2)		19(33.9)		11(19.6)		12(19.4)	
No	56(47.5)	24(38.7)		29(46.8)		15(24.2)		19(33.9)	
**Mother’s BMI, *n* (%)**			0.15		0.25		0.31		0.003
Normal	25(21.2)	10(40.0)		9(36.0)		7(28.0)		3(12.0)	
Overweight	47(39.8)	10(21.3)		16(34.0)		7(14.9)		8(17.0)	
Obese	46(39.0)	17(37.0)		23(50.0)		12(26.1)		20(43.5)	

**Notes.**

a Data from a study of metabolic syndrome in Hispanic children at a community health center in Johnson City, TN, June 2015–June 2016.

b Screen time: includes time watching TV and videos.

c *p*-value from chi-squared or Fisher’s exact test.

Abbreviations PA Physical Activity BMI Body Mass Index BP Blood Pressure >2 h TV/day Greater than an average of 2 h of watching TV or videos in the past 30 days <3 Days PA/Wk Less than 3 days of physical activity for at least 60 min per day in the past 7 days

**Table 3:** Bivariate associations between preadolescents’ physical activity, screen time, blood pressure and body mass index categories (*N* = 118).^a^

Characteristic	Elevated BP *n* (%)		Being overweight/ obese *n* (%)		<3 Days PA/ WK *n* (%)	
		*p*-value		*p*-value		*p*-value
**Blood pressure**						0.30
Normal	//		//		19(23.5)	
Elevated	//		//		12(32.4)	
**Child BMI**		0.046				0.87
Normal	17(24.3)		//		18(25.7)	
Overweight/Obese	20(41.7)		//		13(27.1)	
**Screen time**^b^		0.17		0.85		
>2 h/day	26(28.3)		11(42.3)		//	
<2 h/day	11(42.3)		37(40.2)		//	

**Notes.**

//, Bivariate association is either not of interest or appear on a different row in this same table.

a Data from a study of metabolic syndrome in Hispanic children at a community health center in Johnson City, TN, June 2015–June 2016.

b Screen time: includes time watching TV and videos.

Abbreviations BP Blood Pressure BMI Body Mass Index <3Days PA/WK Less than 3 days of physical activity for at least 60 min per day in the past 7

In adjusted multiple logistic regression, preadolescents of obese mothers were 6.47 times more likely than preadolescents of mothers with normal BMI to engage in less than three days of at least 60 min of vigorous PA per week (95% CI [1.61–26.0]) (Table 4). Preadolescents whose mothers did not engage in moderate PA were 2.92 times more likely to engage in less than three days of at least 60 min of vigorous PA per week (95% CI [1.18–7.24]); and 2.5 times more likely to have elevated BP (95% CI [1.02–4.53]) than preadolescents whose mothers engaged in moderate PA (Table 4).

**Table 4:** Odds ratios and 95% confidence intervals for the associations between maternal body mass index and physical activity and preadolescents’ physical inactivity, being overweight/obese, and elevated blood pressure (*N* = 118).^a^

	<3 days of PA/WK	Being overweight or obese	Elevated BP
	OR (95% CI)^b^	OR (95% CI)^b^	OR (95% CI)^c^
**Age group**			
2–5 vs 6–10 years	0.24(0.04–1.33)	0.90(0.40–2.02)	4.45(1.68–11.78)
**Male vs female**	0.70(0.27–1.84)	1.32(0.61–2.87)	2.06(0.82–5.21)
**Mother’s education**			
<9th grade (ref)			
9–11th grade	0.24(0.04–1.33)	0.74(0.24–2.25)	0.75(0.21–2.68)
High school/GED	2.44(0.77–7.79)	0.83(0.33–2.13)	0.548(0.17–1.68)
≥Some college	1.02(0.21–5.01)	0.83(0.223.13)	1.14(0.25–5.20)
**Moderate PA by mother**			
Yes(ref)			
No	2.92(1.18–7.24)^*^	1.77(0.82–3.83)	2.50(1.02–4.53)^*^
**Mother’s BMI**			
Normal(ref)			
Overweight	1.20(0.28–5.27)	na	0.41(0.13–1.27)
Obese	6.47(1.61–26.0)^**^	na	0.70(0.24–2.02)

**Notes.**

a Data from a study of metabolic syndrome in Hispanic children at a community health center in Johnson City, TN, June 2015–June 2016.

b Adjusted for mother’s education, child’s age, sex.

c Adjusted for mother’s education, child’s sex, child age, child BMI, and child PA.

Odds ratios were calculated from multiple logistic regression models.

Abbreviations GED General Education Development PA Physical Activity BMI Body Mass Index BP Blood Pressure <3 Days PA/WK Less than 3 days of physical activity for at least 60 min per day in the past 7 days na not tested in multiple logistic regression because alpha of univariate association between variables was ≥0.20

* *p*-value < 0.05.

** *p*-value < 0.01.

## Discussion

In this study, we found that about four out of every ten preadolescents were overweight or obese (40.7%), and about three out of every ten had elevated BP (31.4%). The study sample’s prevalence of being overweight/obese is similar to national estimates for Hispanic children (Ogden et al., 2014). Estimates of the prevalence of elevated BP in children are few and difficult to compare. In a large cohort study of 3-to-18-year-old children presenting for well-child care, Hansen, Gunn & Kaelbar (2007) determined the prevalence of elevated BP to be 7.0%. A study by Ma, Zhang & Xi (2016) using NHANES 2013–2014 data reported the prevalence of elevated BP in 8-to-17-year-olds to be 7.0%. Kit et al. (2015) also using NHANES data estimated the prevalence of elevated BP in 8-to-17-year-old children to be 11.0% in 2011–2012. The prevalence of elevated BP in this study exceeds estimates from these previous studies. One explanation is the low socioeconomic status of participants. About 89% of mothers had only a high school education or less. Low socioeconomic status is associated with significantly higher obesity and hypertension (Brummett et al., 2011; Van Den Berg et al., 2013).

This study also found that preadolescents of obese mothers were 6.47 times more likely than preadolescents of mothers with normal BMI to engage in less than three days of at least 60 min of vigorous PA per week; and preadolescents whose mothers did not engage in moderate PA were 2.92 times more likely to engage in less than three days of at least sixty minutes of vigorous PA than preadolescents whose mothers engaged in moderate PA. Our findings comport with previous studies which have found strong mother-child correlations in PA and a strong association between maternal obesity and child PA. In a study of 3-to-5-year-old Hispanic preadolescents and their mothers at a local community center in Nashville, TN, Ruiz et al. (2011) observed strong mother-child correlation in sedentary behavior and moderate PA; Fuemmeler, Anderson, and Masse (2011) found a high correlation of moderate-to-vigorous PA (MVPA) between mothers and their preadolescents. Rosendranz & Dzewaltowski (2011) found that mother-child shared PA was negatively associated with child BMI percentile, and that maternal BMI was positively correlated with child BMI. The same study evaluated the effects of PA-related-parenting behaviors such as encouragement, transporting the child for PA and watching the child do PA. Of all PA-related-parenting behaviors, PA encouragement had the strongest correlation with child PA.

We also found that preadolescents whose mothers did not engage in moderate PA were 2.5 times more likely to have elevated BP than preadolescents whose mothers engaged in moderate PA. Physical inactivity and overweight are risk factors for elevated BP in preadolescents (Ewald & Haldeman, 2016; Gopinath et al., 2014; Leary et al., 2008). However, after controlling for child PA, and BMI, preadolescents whose mothers did not engage in moderate PA remained significantly more likely to have elevated BP than preadolescents whose mothers engaged in moderate PA. To the best of our knowledge, this is the first study to report a significant association between maternal PA and child BP in Hispanic preadolescents, independent of child’s BMI and PA.

This study has some limitations. The sample size was relatively small; hence the study may have lacked power to reach significance for some of the associations. Second, being a cross-sectional study of predominantly Mexican-Americans, our findings may not be generalizable to all Hispanic preadolescents and do not indicate causality. Longitudinal studies are needed for further investigating the correlation between maternal and preadolescent factors examined in this study. Third, our inability to control for diet may potentially confound our findings. However, these weaknesses should be weighed against the strengths of this study. To the best of our knowledge, this is the first study which has assessed the prevalence and sociodemographic correlates of elevated BP in Hispanic preadolescents in Tennessee.

## Conclusions

The prevalence of overweight or obesity and elevated BP among Hispanic preadolescent subjects, especially in 2-to-5-year-olds was higher than expected based on estimates from national studies (Kit et al., 2015; Ma, Zhang & Xi, 2016). Low maternal PA was associated with elevated BP and lower PA in preadolescents; likewise, maternal obesity was associated with lower PA in preadolescents. Our findings suggest the need for public health interventions that would encourage mothers to increase physical activity and improve weight control as a means for reducing obesity and hypertension in Hispanic preadolescents.

## Additional Information and Declarations

### Data Availability

The following information was supplied regarding data availability: The corresponding author, Dr. Arsham Alamian (alamian@etsu.edu), is the PI of the project from which this manuscript has been generated. The data were obtained as part of an externally funded research grant by the State of Tennessee on an at risk vulnerable population of Northeast Tennessee. The PI holds the original crude data and is obligated by the State of Tennessee and East Tennessee State University to keep the data confidential and locked. However, The PI is happy to share the data with any reputable author who requests the data and conform with the data confidentiality standards that are in place by signing a confidentiality data release form.
